# EVALUATING ITEM NONRESPONSE IN A LIFE HISTORY CALENDAR: AN ANALYSIS OF MEMORY EFFECTS

**DOI:** 10.1093/geroni/igz038.3138

**Published:** 2019-11-08

**Authors:** Mengyao Hu, Roberto Melipillán, Xinyu Zhang, Jacqui Smith

**Affiliations:** 1 University of Michigan, Ann Arbor, Michigan, United States; 2 UNIVERSIDAD DEL DESARROLLO, Concepción, Chile

## Abstract

Memory decline contributes to response inaccuracy and can produce item missing data, especially in retrospective surveys with older adults. Event history calendars, or the life grid approaches, are commonly used to obtain retrospective life history data. As indicated in previous literature, this approach can assist respondents’ memory retrieval. Despite its wide use, the important issue of item nonresponse due to memory effects in life grid questions has received little attention. Autobiographical memory (AM) research has shown that there are two interconnected long-term memory systems: episodic memories of event details from specific remote times in an individual’s life; and semantic memories of the important facts and themes that define an individual’s life history. Episodic and semantic AM may introduce different levels of difficulty in retrieving memory and thus contribute to different levels of missing data. This study examines the effects of both item-level predictors (e.g., types of memories) and respondent-level predictors (e.g., cognitive status, age, and health status) on the likelihood of item missing data in life grid questions. We analyzed missing data in the 2017 Health and Retirement Study (HRS) Life History Mail Survey (n = 3,844), using multilevel logistic regression. The results revealed higher rates of item missing for episodic memories, and that overall respondents’ cognitive status was significantly associated with their likelihood of providing item missing data. Recent residential information was better recalled than childhood information. These results have implications for life course analysis of exposures linked to residential histories.

